# Spatial incoherence-driven optical reconstruction of holograms with observer shift-invariance

**DOI:** 10.1038/s41377-025-01823-z

**Published:** 2025-05-12

**Authors:** Yeo Ju Sohn, Daeho Yang

**Affiliations:** 1 Department of Family Medicine, Ewha Womans University College of Medicine, Seoul, 07804 South Korea; 2 Department of Physics, Gachon University, Seongnam, Gyeonggi-do 13120 South Korea

**Keywords:** Displays, Imaging and sensing

## Abstract

Coherence preserves phase consistency between wavefields, enabling accurate recording and reconstruction in holography. Although recent advances in computational optics have realized holographic data acquisition using incoherent light by computationally retrieving information, optical reconstruction still requires partially coherent light sources. We demonstrate a hologram that reconstructs 3-dimensional distribution utilizing incoherence. By decomposing incoherent light into infinitesimal coherent lights and calculating their propagations, the incoherent sum is optimized to resemble the desired 3-dimensional scene, whereas individual coherent lights reconstruct completely different intensities. Incoherence provides high image quality and a wide eyebox, with the reconstructed intensity remaining shift-invariant under pupil displacement, allowing a 1000-fold expansion of the eyebox. We confirm the shift-invariance through a proof-of-concept experiment and demonstrate real-time synthesis of incoherent holograms using a neural network, significantly reducing computational costs. Our method could inspire new approaches in photonics using incoherent light and be practically adopted in holographic displays.

## Abstract (web summary)

The expanded eyebox of holographic displays has been realized using incoherent light by optimizing the incoherent sum of infinitesimal coherent components, enabling high-quality, shift-invariant reconstruction and real-time synthesis via neural networks.

## Graphical abstract

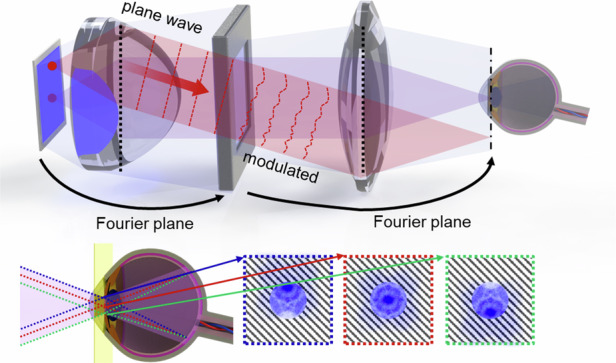

## Introduction

Holography enables recording and reconstructing the wavefield of an object through coherent wavefield interference^1^. The coherent interference of holography allows the recovery of phase information, making it irreplaceable for applications in non-destructive testing^2^, data storage^3^, and biomedicine^4^. Moreover, 3-dimensional (3D) images can be reconstructed through phase information^5^, making holography valuable in microscopy^6^, biological 3D imaging^7^, and 3D object recognition^8^. Since holography relies on interference, coherent light sources are traditionally considered essential for obtaining accurate reconstructions.

However, advanced computational methods have enabled the acquisition of holographic data using incoherent light^9–13^. Unlike traditional coherent holography, which records the wavefront of light, incoherent holography computationally reconstructs 3D location information, reducing speckle noise and minimizing sample damage. For instance, Fresnel incoherent correlation holography (FINCH) adopts self-interference between wavefields with a common optical path to capture interference patterns^11^, while optical scanning holography (OSH) uses active optical heterodyne scanning to record holograms^12^. Furthermore, the complex-valued hologram is extracted without interference by modulating and recording multiple intensities in interferenceless coded aperture correlation holography (I-COACH)^13^.

**Fig. 1:**
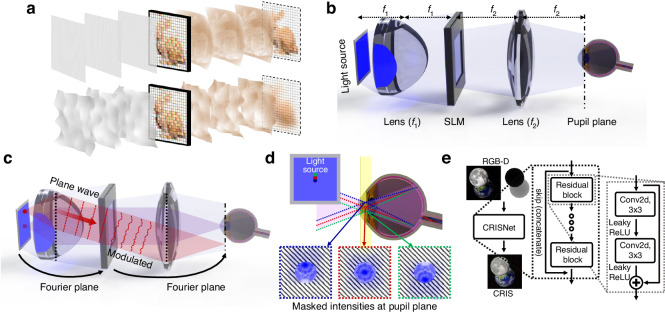
Schematics of CRIS. **a** Reconstruction of hologram using coherent and incoherent light. The consistent phase of a coherent light source enables clear reconstruction, while random phase of an incoherent light source prevents sharp reconstruction. **b** Schematic configuration for implementing CRIS. To realize the incoherent sum of coherently propagated wavefields, the SLM should be located on the Fourier plane of the light source, and the pupil should be near the Fourier plane of the SLM. **c** A point light source corresponds to the plane wave at the SLM and the modulated plane wave is filtered by the pupil aperture. **d** Subset of incoherent light passing through a pupil. Only the light passing through the pupil needs to be considered among the numerous light components to numerically simulate the reconstructed intensity. The yellow plane represents the pupil plane, which is the Fourier plane of the SLM. Each dotted line represents light from a different point in the light source, and each inset shows the corresponding intensity on the pupil plane, masked by the pupil. Due to the optical filtering effect of the pupil, each light reconstructs a different intensity at the retina. **e** Structure of the CRISNet. The network accepts an RGB-D image as an input and returns a modulation for CRIS

Incoherent light sources have been extensively explored not only in holographic data acquisition but also in optical 3D reconstruction to address issues associated with coherent light sources^14–16^. A superluminescent LED is utilized as a partially coherent light source to mitigate unwanted interference and noise^14^. Coherence of the light source is optimized^15^, or multiple sources are employed^16^, to reduce speckle noise and enhance resolution. However, optical reconstruction is limited to partially coherent regimes, as increased incoherence hinders 3D reconstruction by stochastically modulating wavefield^17^ (Fig. 1a).

Here, we demonstrate the optical reconstruction of a hologram based on incoherent light, requiring incoherence instead of coherence. Our method, Coherently-Reconstructed Incoherent Sum (CRIS), optically reconstructs the hologram via the incoherent sum of coherent lights. More specifically, the intensity at the retina is calculated by propagating and accumulating the subset of incoherent light passing through the pupil, while the simulated intensity is optimized to resemble the target 3D scene. Spatial incoherence allows the scene to be viewed from various angles, achieving 3D reconstruction with wide eyebox—characteristics previously thought to be mutually exclusive in holographic and conventional displays^18,19^. We theoretically demonstrate that CRIS remains shift-invariant under pupil displacement and experimentally confirm the shift-invariance. Theoretically, the shift-invariance allows for a 1000-fold expansion of the eyebox, though our proof-of-concept experiment achieved a 32-fold expansion. At the same time, we trained a neural network for real-time synthesis because simulating and optimizing an incoherent sum requires millions of times more computational resources than conventional holograms, making its use in holographic displays impractical. We anticipate our method will enable the widespread adoption of holographic displays by addressing key challenges–high image quality, real-time synthesis, and wide eyebox–without additional equipment, while also inspiring new approaches with incoherent light.

## Results

For coherent light incident on a spatial light modulator (SLM), the scattering angle is determined by its pixel pitch. Consequently, the light is visible only within a small area, known as the eyebox, and even minor eye movements can cause the reconstructed image to disappear. On the other hand, illuminating the SLM with incoherent light, such as that from a large-area LED, introduces a broad range of incident angles. Due to the large incident angle exceeding the diffraction angle of the SLM, the modulated light spread over various output directions, enabling a wide viewing angle regardless of the diffraction angle of the SLM, as seen in conventional displays. However, such displays are not capable of optically reconstructing arbitrary 3D scenes due to the incoherence of the light source (Fig. 1a).

To address the limitation and utilize coherence among incoherent light, we used spatial coherence associated with an individual point light source. A single point light source exhibits spatial coherence, while a spatially incoherent light source can be considered as an ensemble of multiple uncorrelated point sources. By placing the SLM in the Fourier plane of the light source, light from a point forms a plane wave with a specific incident angle at the SLM plane (Fig. 1b). When considering only the light with the specific incident angle at the SLM plane, spatial coherence exists, and the light modulated by the SLM can reconstruct an arbitrary 3D wavefield.

After each modulated plane wave passes through another lens, the modulated light is Fourier transformed at the pupil plane (Fig. 1c). Although each plane wave is modulated by the same SLM pattern, the modulated light passing through the pupil differs due to the filtering effect of the pupil. Since the pupil plane corresponds to the Fourier plane of the SLM, each modulated wave is represented as a Fourier-domain wave on the pupil plane. Each Fourier-domain wave, shifted proportionally to its plane wave angle, is masked by a pupil-defined Fourier filter (Fig. 1d). As a result, each modulated plane wave has different intensities at the retina, and the total intensity can be calculated as the incoherent sum of the individual intensities of the filtered waves. By integrating a subset of incident light imaged by the pupil aperture, the total intensity of CRIS can be expressed as,

1 $${I}_{{\rm{CRIS}}}\left({\vec{r}},z\right)=\frac{1}{{\mathcal{N}}}\sum _{{\vec{k}}_{g}}{\left\vert {\int}_{{\mathcal{A}}}{\bar{U}}({\vec{k}}+{{\vec{k}}}_{g}){e}^{iz\sqrt{{(2\pi /\lambda )}^{2}-{\left\vert {\vec{k}}\right\vert }^{2}}}{e}^{i{\vec{k}}\cdot \vec{r}}d{\vec{k}}\right\vert }^{2}$$

where, ${I}_{{\rm{CRIS}}}({\vec{r}},z)$ is the reconstructed intensity, *z* is the distance along the longitudinal direction, ${\mathcal{N}}$ is a normalization constant, ${\vec{k}}_{g}=({k}_{gx},{k}_{gy})$ is the radial wavevector of the collimated incident light, ${\bar{U}}({\vec{k}})$ is the Fourier transformed function of the SLM modulation $U(\vec{r})$, ${\vec{k}}=({k}_{x},{k}_{y})$ is the radial wavevector of the modulated field, *λ* is the wavelength of the light, and ${\mathcal{A}}$ is the area of the pupil in the Fourier plane. Since the pupil blocks different spatial frequencies of the modulation based on ${\vec{k}}_{g}$, each filtered wavefield represents only a partial frequency region of the modulation. (Fig. 1d). In summary, the reconstructed total intensity for a specific depth can be calculated by accurately accounting for all coherent propagation of these partial modulations.

By introducing the pupil location vector from the center of the eyebox, (*p*_*x*_, *p*_*y*_, *p*_*z*_), it is possible to derive the shift-invariant formula (see “Methods”),

2 $${I}_{{\rm{CRIS}}}({\vec{r}},z,{p}_{x},{p}_{y},{p}_{z})={I}_{{\rm{CRIS}}}\left({\vec{r}},z,0,0,0\right)$$

Equation (2) is valid under the Fresnel approximation, and thus the reconstructed intensity is invariant even if the pupil shifts along all axes within the region where the Fresnel approximation holds. Therefore, if we find a proper Fourier transformed modulation ${\bar{U}}({\vec{k}})$ satisfying ${I}_{{\rm{CRIS}}}({\vec{r}},z)={I}_{{\rm{target}}}$, then we can reconstruct a 3D scene which can be seen everywhere by displaying the modulation $U(\vec{r})$. The shift-invariance can be understood as the cancellation between the grating phase of the incident angle and the shift in the Fourier domain.

When the pupil shifts in the Fourier plane, we can find the specific incident angle at which the light passes through the center of the pupil (Fig. 1c). Under the Fresnel approximation, the corresponding light has the same wavefield as the light with a zero incident angle, except for the oscillating phase induced by the incident angle. The same rationale can be applied to marginal light; therefore, the incoherent sum would be the same as the case when the pupil is at the center. A more rigorous derivation of the shift-invariance of the CRIS, including the shift along the *z*-axis, can be found in the Methods.

To find the modulation satisfying Eq. (1), gradient descent methods can be used. After calculating the right-hand side of Eq. (1) for an arbitrary initial modulation, the difference between the incoherent sum and the target intensity is calculated. The modulation is updated to minimize the difference, and the process is repeated until the predefined number of iterations is reached. To numerically simulate incoherent light propagation, coherent propagations are calculated for hundreds of different incident angles, and all the coherent propagation results are incoherently added (see Supplementary Material). As a result, millions of coherent propagations are required to synthesize a single hologram, making the algorithm impractical for real-time applications.

To overcome the limitation, we trained a neural network (CRISNet) where the model is composed of residual blocks with 3 × 3 convolutional layers (Fig. 1e). To train multi-depth holograms, the incoherent sum is numerically calculated for each depth and compared with the depth-dependent target image. The depth-dependent target image is synthesized by multiplying an all-in-focus image with a mask in which only the pixels at each corresponding depth are nonzero. As a result, the total cost function is given as

3 $${\mathcal{L}}=\sum _{n}M(\vec{r},{d}_{n})\left[{I}_{{\rm{CRIS}}}(\vec{r},{d}_{n})-{I}_{{\rm{target}}}(\vec{r})\right]$$

where, *d*_*n*_ is distance between the *n*th layer and the SLM, $M(\vec{r},{d}_{n})$ is the mask for pixels in the *n*th layer, and ${I}_{{\rm{target}}}(\vec{r})$ is the target intensity.

**Fig. 2:**
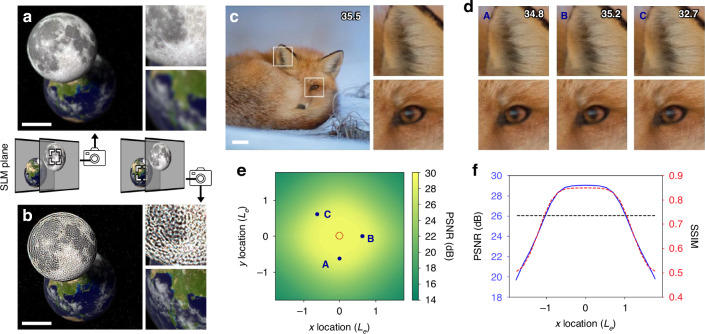
Numerical reconstruction of CRIS. **a**, **b** Numerically reconstructed 3D scene of the CRIS. The moon is on the front plane, while the earth is on the SLM plane. The moon is sharply reconstructed when the focus is on the front plane (**a**), and the earth is sharply reconstructed when the focus is on the SLM plane (**b**). The white horizontal bar represents a scale bar corresponding to 2^∘^ of angular field of view. **c** Numerically reconstructed floating 2D scene to evaluate the image quality metric. The Peak Signal-to-Noise Ratio(PSNR) in dB is marked in the top right corner, and the insets present enlarged images. **d** Enlarged images of numerically reconstructed intensities as a function of the pupil location. The pupil locations of each image correspond to $(0,-{L}_{e}/\sqrt{2})$, $({L}_{e}/\sqrt{2},0)$, and $(-{L}_{e}/\sqrt{2},{L}_{e}/\sqrt{2})$. **e** Average PSNRs as a function of the pupil location, with the location normalized by the half eyebox length, *L*_*e*_. The red dotted circle represents the non-expanded eyebox of conventional holograms (see Supplementary Material). **f** Average PSNRs and Structural Similarity Index Measures(SSIMs) as a function of the pupil location along the *x*-axis. The blue solid line represents the average PSNRs and the red dashed line represents the average SSIMs. The black dotted line represents the—3dB line from the maximum PSNR, which corresponds to half of the signal-to-noise ratio

Figure 2 presents the numerical reconstruction results of the CRIS. The CRIS is synthesized to reconstruct the scene, with the moon located at the floating plane in front of the SLM and the earth located at the SLM plane. To numerically simulate CRIS, the virtual image of the Earth is assumed to be located at infinity, corresponding to 0 diopters, while the virtual image of the Moon is assumed to be located 67 cm from the pupil, corresponding to 1.5 diopters. The numerical reconstruction presents that the earth appears blurred while the moon remains sharp when the focus is on the front plane, implying 3D reconstruction (Fig. 2a). In contrast, the earth becomes sharp when the focus is on the SLM plane, although the defocus blur of the moon is not natural (Fig. 2b). The defocus blur can be modified by adopting additional loss functions in sake of reducing image quality^20,21^ (See Supplementary Material).

Given that most image quality metrics are designed for 2D images, we utilized a 2D image dataset, the DIV2K validation dataset^22^, to quantitatively analyze the image quality of the CRIS. We assumed that the virtual images were positioned 1.5 diopters from the SLM plane to simulate floating objects, where 1.5 diopters correspond to a typical interaction length of 67 cm, approximately the length of a human arm. One of the numerically reconstructed floating images, for the case where the pupil is at the center of the eyebox, is presented in Fig. 2c. Additional reconstruction intensities for different target images are shown in Fig. 7.

To demonstrate the shift-invariance of the CRIS, the same image is numerically reconstructed at different locations of the eyebox (Fig. 2d). According to the theoretically derived formula (see “Methods”), the half length of the eyebox along the *x*-and *y*-axes is given as ${L}_{e}={(8\lambda {f}^{2}/D)}^{\frac{1}{4}}$, where *D* is the diopter. Each point in Fig. 2d corresponds to the points $(0,-{L}_{e}/\sqrt{2})$, $({L}_{e}/\sqrt{2},0)$, and $(-{L}_{e}/\sqrt{2},{L}_{e}/\sqrt{2})$. To visualize the distribution of image quality, the CRIS is synthesized for all images in the dataset and the PSNR values are averaged for each pupil location through the numerical reconstruction. Figure 2e presents the average PSNR as a function of the *x* and *y* location in a unit of *L*_*e*_. As the shift-invariance does not hold beyond the valid range of the Fresnel approximation, the average PSNR decreases near the distance *L*_*e*_. A cross-sectional graph confirms that the full width at half maximum (FWHM) of the PSNR is 2*L*_*e*_, indicating agreement between the numerical simulation results and the theoretically derived formula within 1.6% (Fig. 2f). The average PSNR for the pupil at the center of the eyebox is 29 dB and that for the pupil at the distance *L*_*e*_ is 26.3 dB.

One of the notable points of the eyebox length is that the eyebox length does not depend on the pixel pitch of an SLM. Therefore, by increasing the focal length of the lens, it is possible to increase the eyebox while sustaining the field of view by using an SLM with a larger physical size. Another important point is that the eyebox length decreases as the diopter of the hologram increases. However, increasing the diopter by a factor of two only results in a 15% decrease in the eyebox length, as it is inversely related to the 1/4 power of the diopter. (See “Methods”)

Addressing the etendue or eyebox limitations of holographic displays has long been studied. For instance, to increase the etendue, a scattering mask is placed while the scattering noise is reduced by optimizing the wavefield^23^ and adopting a neural etendue expander^24^. Moreover, a photon sieve is utilized to enhance the viewing angle^25^. However, supporting high image quality for every pupil location remains challenging, whereas CRIS can achieve the wide eyebox and high image quality with a single hologram.

**Fig. 3:**
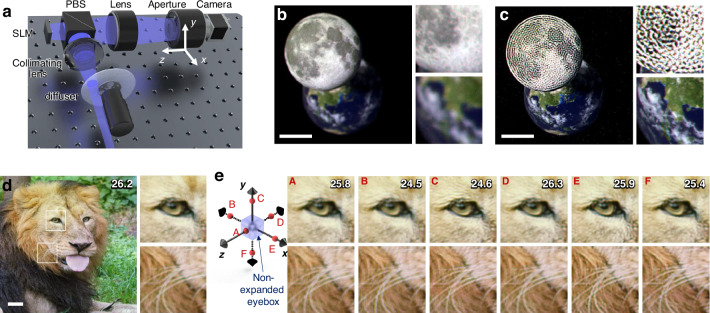
Optical reconstruction of CRIS. **a** Schematic image of the experimental setup. A collimated RGB laser beam is scattered by a diffuser and collimated by another lens. An amplitude-only SLM with a polarizing beam splitter cube (PBS) is used to reconstruct the hologram. The camera with a lens and an aperture captures optically reconstructed holograms while its location is adjusted along the *x*, *y*, and *z* directions. The aperture corresponds to the pupil aperture of an eye and thus the aperture and the camera effectively behave as a human eye. Optically reconstructed images of the moon and earth CRIS are shown when the camera focus is on the front plane (**b**) and the SLM plane (**c**). The white horizontal bar represents a scale bar corresponding to 2^∘^ of angular field of view. **d** Optically reconstructed 2D scene floating 1.5 diopters from the SLM plane. The PSNR in dB is marked in the top right corner, and the insets present enlarged images. **e** Enlarged images of optically reconstructed intensities as a function of the pupil location. The locations are marked on the axes, with the location normalized by the lengths of the non-expanded eyebox

To confirm the shift-invariance of the CRIS, optical reconstruction (i.e., experimental reconstruction) is performed (Fig. 3). Although spatial coherence should be suppressed in realizing the CRIS, sustaining temporal coherence is ideal for providing high image quality. To create the light source assumed in the theoretical calculation, red, blue, and green lasers are coupled to a single-mode fiber, and a rotating diffuser is employed to suppress spatial coherence. Even though temporally coherent light was used in the experiment, image quality is anticipated to be sustained without temporal coherence (see Supplementary Material). An amplitude-only SLM is used to optically reconstruct the CRIS whereas the CRIS is synthesized to be represented solely by amplitude. Contrary to conventional holograms, the Fourier plane is filled with light from various incident angles, making the use of a Fourier filter impossible. Although not explicitly stated in the numerical reconstruction, all the reconstructed images and benchmarks are based on the amplitude-only representation.

With the same 3D scene presented in the numerical reconstruction, the optical reconstruction shows the sharp moon and the blurred earth when the camera focus is on the front plane, indicating successful reconstruction of floating objects (Fig. 3b). When the camera focus is on the SLM plane, the earth becomes sharp (Fig. 3c). The suppressed spatial coherence prevents speckle noise induced by laser coherence and dust, which are the major sources of noise in conventional holograms, resulting in high image quality. Additional 3D scenes for different pupil locations are shown in Fig. 8.

**Fig. 4:**
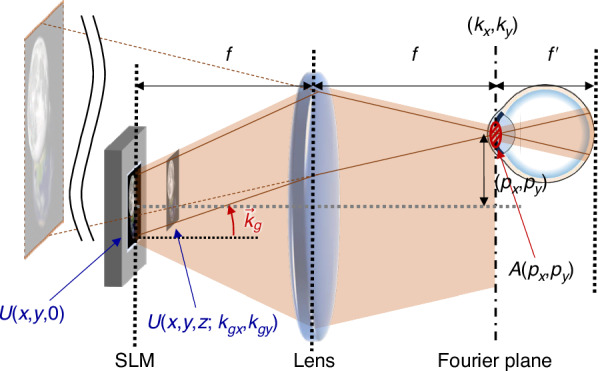
Schematics for calculating pupil shift-invariance. For the pupil location (*p*_*x*_, *p*_*y*_), the virtual image of the reconstructed hologram remains fixed relative to the eye. The marked coordinates and variables are consistent with Eqs. (M1), (M2), and (M3)

To assess image quality metrics based on the aperture location, we optically reconstruct a 2D floating image and evaluate the PSNR as the aperture location varies. Figure 4d shows the optically reconstructed image with enlarged insets when the aperture is at the center of the eyebox. Even when the aperture is moved beyond the boundaries of the non-expanded eyebox, the image quality remains similar (Fig. 4e). Furthermore, shifting the aperture along the *z*-axis does not affect image quality or field of view, contrary to conventional holograms. In other words, we experimentally confirmed the shift-invariance, which theoretically allows a 1000-fold expansion of the eyebox, where the theoretical eyebox expansion ratio is calculated by comparing 2*L*_*e*_ and the conventional eyebox (see “Methods”). Since the restricted numerical aperture of our setup limits the incident angle of light, the measured eyebox volume is 4.5 × 4.5 × 12 mm^3^, representing a 32-fold expansion. Nonetheless, the experimental demonstration of the shift-invariance suggests that the eyebox can be further increased by adopting optics with a larger numerical aperture (See “Methods”).

Although the CRIS can resolve the eyebox problem, there still remain limitations related to depth representation, including the absence of motion parallax and unnatural defocus blur. Since the CRIS provides the same 3D scene regardless of pupil locations, motion parallax does not exist in the CRIS. The absence of motion parallax could be an issue in flat-panel holographic displays; however, it is not problematic for near-eye displays with head motion-induced parallax. Considering that holographic displays for near-eye displays have been extensively studied recently^24,26,27^ due to the augmented, virtual, and mixed reality applications, CRIS offers a promising solution for these contexts, particularly for supporting accommodation effect.

## Discussion

Holographic displays have long been considered to be the future of displays due to the advantage of providing photorealistic 3D scenes without vergence-accommodation conflicts^28^. However, to deliver immersive experiences, holographic displays should match conventional 2D displays in every aspect while also reconstructing 3D scenes. Although recent advancements have addressed issues related to high computational costs and low image quality, a small eyebox problem still remains. Despite various proposals for eyebox expansion, every method has its own challenges, including low image quality, double vision, or low responsiveness, leading to less immersive experiences compared to conventional displays^29,30^. Our method addresses the limited eyebox problem while providing coherent-artifact-free reconstruction like conventional displays, offering immersive experiences.

Recently, ordinary displays have been utilized to reconstruct holograms without relying on coherent light sources^31^. While both the ordinary display approach and our approach utilize incoherent light sources, the two methods have distinct goals. The former approach focuses on utilizing ordinary displays under general conditions without constraints; however, a wide eyebox is not expected because the optimized intensity is limited to a fixed camera. In contrast, CRIS prioritizes achieving eyebox expansion by applying specific constraints to the positions of the light source and SLM, while also utilizing the filtering effect of the pupil aperture.

Unnatural defocus blur is a common issue in holographic displays, manifesting as both weak defocus blur and defocus blur with speckles. In holograms with smooth phase, suppressed light scattering provides high image quality but leads to weak defocus blur^32^. Conversely, in holograms with non-smooth phase, increased light scattering induces high-contrast speckles in out-of-focus areas, disrupting the correct perception of depth cues^33^. Although the CRIS provides natural defocus blur for objects on the SLM plane, the defocus blur for objects at other planes remains unnatural. Various methods can address the unnatural defocus blur^20,34^, and employing these methods can relieve the issue (See Supplementary Material).

In summary, we proposed a new hologram, CRIS, and theoretically derived the shift-invariance property. The proof-of-concept experiment demonstrates that the shift-invariance holds for all axes and reconstructing a 3D scene is possible. The CRIS not only inspires new approaches using incoherent light but also resolves key challenges of holographic displays such as eyebox limitations, image quality, real-time synthesis, and responsiveness. We expect the CRIS to promote the widespread use of holographic displays by providing 3D reconstruction with the enhanced properties comparable to those of 2D displays. Moreover, the incoherent optical reconstruction will enhance the understanding of incoherent light in holography and enable various applications based on its accurate consideration.

## Materials and methods

### Shift-invariance property under Fresnel approximation

According to the Angular Spectrum Method (ASM), the Fourier-transformed wavefield ${\bar{U}}$ can be expressed as ${\bar{U}}({k}_{x},{k}_{y},z)={\bar{U}}({k}_{x},{k}_{y},0){e}^{iz\sqrt{{k}^{2}-{k}_{x}^{2}-{k}_{y}^{2}}}\approx {\bar{U}}({k}_{x},{k}_{y},0){e}^{ikz\left(1-\frac{{k}_{x}^{2}+{k}_{y}^{2}}{2{k}^{2}}\right)}$, where we used the Fresnel approximation. The propagated wavefield *U*(*x*, *y*, *z*) can be expressed as,

M1 $$\begin{array}{lll}U(x,y,z)\,=\,\displaystyle\int\int\bar{U}({k}_{x},{k}_{y},0)\\\qquad\qquad \,\times {e}^{ikz\left(1-\frac{{k}_{x}^{2}+{k}_{y}^{2}}{2{k}^{2}}\right)}{e}^{i{k}_{x}x+i{k}_{y}y}d{k}_{x}d{k}_{y}\end{array}$$

By considering spatially incoherent light as incoherent sum of numerous coherent light components, Eq. (M1) can be used to express a hologram reconstructed by coherent light. To consider coherent light with different incident angles, the grating phase, ${e}^{-i{k}_{gx}x-i{k}_{gy}y}$, should be applied to the wavefield before propagation, *U*(*x*, *y*, 0). As a result, Eq. (M1) can be modified to consider the propagated wavefield with different propagation angle, (Fig. 4)

M2 $$\begin{array}{lll}U(x,y,z;{k}_{gx},{k}_{gy})\,=\,\displaystyle\int\int\bar{U}({k}_{x}+{k}_{gx},{k}_{y}+{k}_{gy},0)\\\qquad\qquad\qquad\qquad \,\times {e}^{ikz\left(1-\frac{{k}_{x}^{2}+{k}_{y}^{2}}{2{k}^{2}}\right)}{e}^{i{k}_{x}x+i{k}_{y}y}d{k}_{x}d{k}_{y}\end{array}$$

where the grating phase, ${e}^{-i{k}_{gx}x-i{k}_{gy}y}$, is removed by shifting the Fourier-transformed wavefield to $\bar{U}({k}_{x}+{k}_{gx},{k}_{y}+{k}_{gy},0)$.

A pupil of an eye behaves as an aperture, so only a limited area, ${\mathcal{A}}({p}_{x},{p}_{y})$, should be integrated in Eq. (M2) to numerically reconstruct a visible intensity. By integrating the holograms reconstructed by coherent light components with different incident angles, the incoherent sum can be expressed as

M3 $$\begin{array}{lll}I(x,y,z,{p}_{x},{p}_{y})\,=\,\displaystyle\frac{1}{{\mathcal{N}}}\mathop{\sum}\limits _{{k}_{gx},{k}_{gy}}\left\vert \displaystyle\int{\int}_{{\mathcal{A}}({p}_{x},{p}_{y})}\bar{U}({k}_{x}+{k}_{gx},{k}_{y}+{k}_{gy},0)\right.\\\qquad\qquad\qquad\;\; \,{\left.\times {e}^{ikz\left(1-\frac{{k}_{x}^{2}+{k}_{y}^{2}}{2{k}^{2}}\right)}{e}^{i{k}_{x}x+i{k}_{y}y}d{k}_{x}d{k}_{y}\right\vert }^{2}\end{array}$$

where ${\mathcal{A}}({p}_{x},{p}_{y})$ denotes the area depending on the position of the pupil. Here we assumed the pupil as a circle with the center (*p*_*x*_, *p*_*y*_) and radius *p*_*r*_.

To explicitly represent the dependency of integration area on the pupil location, we rewrite Eq. (M3) by shifting *k*_*x*_ and *k*_*y*_. Considering *f* is the focal length of the lens used to make the spatial Fourier transform, we can define the shifted wavevectors as, ${k}_{x}\equiv {k}_{x}^{{\prime} }+{k}_{px}$, ${k}_{y}\equiv {k}_{x}^{{\prime} }+{k}_{py}$, *k*_*px*_ ≡ *kp*_*x*_/*f*, *k*_*py*_ ≡ *kp*_*y*_/*f*. The re-written equation is given as

M4 $$\begin{array}{lll}I(x,y,z,{p}_{x},{p}_{y})\,=\,\displaystyle\frac{1}{{\mathcal{N}}}\mathop{\sum}\limits _{{k}_{gx},{k}_{gy}}\left\vert \displaystyle\int{\int}_{{\mathcal{A}}(0,0)}\bar{U}({k}_{x}^{{\prime} }+{k}_{px}+{k}_{gx},{k}_{y}^{{\prime} }+{k}_{py}+{k}_{gy},0)\right.\\\qquad\qquad\qquad\quad \,{\left.\times {e}^{ikz\left(1-\frac{{({k}_{x}^{{\prime} }+{k}_{px})}^{2}+{({k}_{y}^{{\prime} }+{k}_{py})}^{2}}{2{k}^{2}}\right)}{e}^{i({k}_{x}^{{\prime} }+{k}_{px})x+i({k}_{y}^{{\prime} }+{k}_{py})y}d{k}_{x}^{{\prime} }d{k}_{y}^{{\prime} }\right\vert }^{2}\end{array}$$

Since the summation range of *k*_*gx*_(*k*_*gy*_) is from negative infinity to positive infinity, we can arbitrarily shift *k*_*gx*_(*k*_*gy*_), and thus *k*_*gx*_(*k*_*gy*_) can be replaced with *k*_*gx*_ − *k*_*px*_(*k*_*gy*_ − *k*_*py*_). After exchanging *k*_*gx*_(*k*_*gy*_) and performing simple calculation yields the following equation,

M5 $$\begin{array}{lll}I(x,y,z,{p}_{x},{p}_{y})\,=\,\displaystyle\frac{1}{{\mathcal{N}}}\sum _{{k}_{gx},{k}_{gy}}\left\vert \int{\int}_{{\mathcal{A}}(0,0)}\bar{U}({k}_{x}^{{\prime} }+{k}_{gx},{k}_{y}^{{\prime} }+{k}_{gy},0)\right.\\\qquad\qquad\qquad\quad \,{\left.\times {e}^{ikz\left(1-\frac{{k}_{x}^{{\prime} 2}+{k}_{y}^{{\prime} 2}}{2{k}^{2}}\right)}{e}^{i{k}_{x}^{{\prime} }\left(x-\frac{z{k}_{px}}{k}\right)+i{k}_{y}^{{\prime} }\left(y-\frac{z{k}_{py}}{k}\right)}d{k}_{x}^{{\prime} }d{k}_{y}^{{\prime} }\right\vert }^{2}\end{array}$$

During the calculation, the term ${e}^{-ikz\left(\frac{{k}_{px}^{2}}{2{k}^{2}}+\frac{{k}_{py}^{2}}{2{k}^{2}}\right)}{e}^{i{k}_{px}x+i{k}_{py}y}$ is removed because it does not include ${k}_{x}^{{\prime} }$ or ${k}_{y}^{{\prime} }$ and the pure phase term can be removed in the calculation of the absolute square.

To make the result clearer, *x*(*y*) is exchanged to $x+\frac{z{k}_{px}}{k}$($y+\frac{z{k}_{py}}{k}$),

M6 $$\begin{array}{lll}\,I\left(x+\frac{{p}_{x}}{f}z,y+\displaystyle\frac{{p}_{y}}{f}z,z,{p}_{x},{p}_{y}\right)\\\;\; \,=\displaystyle\frac{1}{{\mathcal{N}}}\mathop{\sum}\limits _{{k}_{gx},{k}_{gy}}\left\vert \int{\int}_{{\mathcal{A}}(0,0)}\bar{U}({k}_{x}^{{\prime} }+{k}_{gx},{k}_{y}^{{\prime} }+{k}_{gy},0)\right.\\\;\; \,{\left.\times {e}^{ikz\left(1-\frac{{k}_{x}^{{\prime} 2}+{k}_{y}^{{\prime} 2}}{2{k}^{2}}\right)}{e}^{i{k}_{x}^{{\prime} }x+i{k}_{y}^{{\prime} }y}d{k}_{x}^{{\prime} }d{k}_{y}^{{\prime} }\right\vert }^{2}\\\;\; \,=I\left(x,y,z,0,0\right)\end{array}$$

The right-hand side of Eq. (M6), $I\left(x,y,z,0,0\right)$, does not depend on *p*_*x*_ and *p*_*y*_ while the left-hand side term depends on them. Equation (M6) can be rewritten as a form of

M7 $$I\left(x,y,z,{p}_{x},{p}_{y}\right)=I\left(x-\frac{{p}_{x}}{f}z,y-\frac{{p}_{y}}{f}z,z,0,0\right)$$

Although the reconstructed intensity is shifted by $(\frac{{p}_{x}}{f}z,\frac{{p}_{y}}{f}z)$ according to Eq. (M7), the intensity seen by an eye is not shifted because the eye is also shifted. Such a condition can be easily found by drawing the virtual image optically reconstructed by the lens (Fig. 4). In other words, the observed intensity of a hologram reconstructed by a spatially incoherent light does not depend on the pupil location and thus the hologram can be seen everywhere.

### Generalization of shift-invariance along *z*-axis

**Fig. 5:**
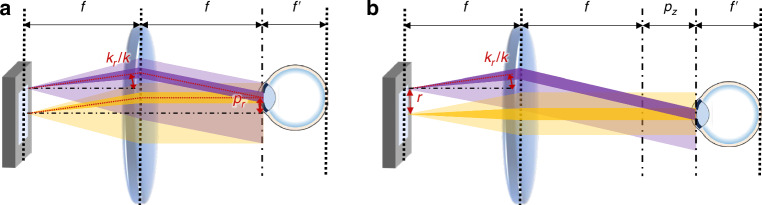
Schematics for calculating effective eyebox. **a** For the radial pupil shift, the radial wavevector of the light passing through the pupil is given as a function of the pupil location. **b** *p*_*z*_ refers to the pupil shift along the *z*-axis from the Fourier plane. For the *z*-axis pupil shift, the radial wavevector of the light passing through the pupil is given as a function of the pupil location and the field location

To account for the pupil shift along the *z*-axis, we need to calculate the complete optical propagation from the SLM to the retina. The propagation can be calculated using the following procedure (Fig. 5b).

1. Propagate for a distance *f*
2. Phase altered by a lens with the focal length *f*
3. Propagate for a distance *f* + *p*_*z*_
4. Phase altered by an eye lens with the focal length ${f}^{{\prime} }$
5. Propagate for a distance ${f}^{{\prime} }$

After simple calculations under Fresnel approximation, the wavefield at the retina is given as,

M8 $$\begin{array}{lll}\,U\left(x,x,f+f+{p}_{z}+{f}^{{\prime} }\right)\\ \,=\,\displaystyle\frac{1}{{{\mathcal{N}}}_{0}}\displaystyle\int\int\,{e}^{-i{k}_{x}x\left(\displaystyle\frac{f}{{f}^{{\prime} }}\right)-i{k}_{y}y\left(\displaystyle\displaystyle\frac{f}{{f}^{{\prime} }}\right)}\bar{U}\left({k}_{x},{k}_{y},0\right)d{k}_{x}d{k}_{y}\end{array}$$

where, ${{\mathcal{N}}}_{0}$ is the normalization constant. The derivation of Eq. (M8) is provided in the Supplementary Material. The right-hand side of Eq. (M8) remains unaffected by the shift of the pupil along the *z*-axis, *p*_*z*_, thus resulting in shift-invariant intensity formed at the retina. The only alteration is the magnification determined by the ratio of focal lengths, $\frac{f}{{f}^{{\prime} }}$. In summary, by combining the result of the previous subsection and Eq. (M8), we can get the following shift-invariant property,

M9 $$I(x,y,z,{p}_{x},{p}_{y},{p}_{z})=I\left(x,y,z,0,0,0\right)$$

Although we rigorously derived the shift-invariance of CRIS, the shift-invariance along the *x*, *y*, and *z* axes is not unique property of our optical system. For example, a lens with an object placed at its focal length also exhibits shift-invariance, as the virtual image forms at infinity and remains unchanged regardless of eye movement due to negligible parallax. Our study, however, demonstrates that a 3D scene can still be reconstructed even when the virtual image of the SLM is at infinity, enabled by wavefield optimization through the filtering effect of the pupil.

### 3-dimensional eyebox size

According to Eq. (M9), the reconstructed intensity does not differ depending on the location of the pupil. However, the fundamental limitation of the CRIS is the Fresnel approximation, and the eyebox is limited by the valid area of the Fresnel approximation. If we consider a plane wave with a radial wavevector, (*k*_*x*_, *k*_*y*_), at the SLM, then the condition for the Fresnel approximation can be expressed as

M10 $$\frac{d}{8\lambda }{\left(\frac{{k}_{r}}{k}\right)}^{4}\ll 1$$

where, ${k}_{r}=\sqrt{{k}_{x}^{2}+{k}_{y}^{2}}$, *d* is the propagation distance, *λ* is the wavelength of the light, and *k* is the magnitude of the wavevector. More specifically, the expansion of the eye box of the CRIS originates from the cancellation of the grating phase from the incident angle by the shift in the Fourier domain. However, the large incident angle breaks the approximation $d\sqrt{{k}^{2}-{k}_{r}^{2}}\approx kd\left(1-\frac{{k}_{r}^{2}}{2{k}^{2}}\right)$, and the cancellation becomes imperfect.

The plane wave with the radial wavevector, (*k*_*x*_, *k*_*y*_), would be focused at the point (*fk*_*x*_/*k*, *fk*_*y*_/*k*) on the Fourier plane and the light is filtered by the pupil (Fig. 5a). By assuming that the radius of the pupil is smaller than the distance between the optical axis and the center of the pupil, we can approximate all the radial wavevectors of the plane wave light passing through the pupil as (*k*_*x*_, *k*_*y*_). It is simple to show that the radial wavevector magnitude of the light passing through the pupil is given as,

M11 $$\frac{{k}_{r}}{k}=\frac{1}{f}\sqrt{{{p}_{x}}^{2}+{{p}_{y}}^{2}}$$

where the pupil position is assumed as (*p*_*x*_, *p*_*y*_, 0).

For the case where the pupil is shifted along the *z*-axis, *k*_*r*_ depends on a distance from the SLM center, *r*. (Fig. 5b) The wavevector magnitude can be calculated as $\frac{{k}_{r}}{k}=\frac{r{p}_{z}}{{f}^{2}}$, and the maximum wavevector magnitude is at the edge of the field of view (FoV). By rewriting in terms of FoV, the maximum of the wavevector magnitude is given as

M12 $$\frac{{k}_{r{\rm{MAX}}}}{k}\approx \frac{1}{f}{p}_{z}\frac{{\rm{FoV}}}{2}$$

By combining Eq. (M11) and Eq. (M12), the eyebox relation can be summarized as,

M13 $$\frac{{k}_{r{\rm{MAX}}}}{k}\approx \frac{1}{f}\sqrt{{{p}_{x}}^{2}+{{p}_{y}}^{2}+{p}_{z}^{2}{\left(\frac{{\rm{FoV}}}{2}\right)}^{2}}$$

According to Eq. (M10) and Eq. (M13), the eyebox can be written as,

M14 $$\frac{1}{f}\sqrt{{{p}_{x}}^{2}+{{p}_{y}}^{2}+{p}_{z}^{2}{\left(\frac{{\rm{FoV}}}{2}\right)}^{2}} < {\left(\frac{8\lambda }{d}\right)}^{1/4}\approx {\left(\frac{8\lambda }{D{f}^{2}}\right)}^{1/4}$$

where, *D* ≈ *d*/*f*^2^ is the depth of the hologram in diopter and the condition “much smaller” is adjusted to “smaller” to account for the fourth power of *k*_*r*_/*k*. The important points of Eq. (M14) are that the eyebox of the CRIS is given as an ellipsoid and its volume depends on the depth of the hologram.

By restricting pupil shift along the *x*-axis, i.e., *p*_*y*_ = 0, *p*_*z*_ = 0, the length of the eyebox along the *x*-axis can be calculated as $| {p}_{x}| < {\left(\frac{8\lambda {f}^{2}}{D}\right)}^{1/4}$. As a result, the half eyebox length along the *x*-and *y*-axes is given as,

M15 $${L}_{e}\equiv {\left(\frac{8\lambda {f}^{2}}{D}\right)}^{1/4}$$

Considering the parameters used in the numerical reconstruction, the eyebox length 2*L*_*e*_ is 12 mm, which is nearly the same as the FWHM of the PSNR, 12.2 mm, with an error of 1.6%. The slight difference can be attributed to the fact that the boundary of the approximation is not well-defined. Interestingly, the eyebox does not depend on the pixel pitch or the number of pixels of the display. Under typical condtions, the eyebox length of a glass-type display (*f* = 20 mm, *λ* = 515 nm, *D* = 1.5 diopters) is 11.5 mm, while that of a flat-panel-type display (*f* = 1000 mm, *λ* = 515 nm, *D* = 1.5 diopters) is 81.4 mm. With our numerical and experimental parameters, the theoretical eyebox volume calculated from Eq. (M14) is 3800 mm^3^, while that of conventional holograms is 3.97 mm^3^, resulting in an enhancement of ~1000 times.

According to Eq. (M15), the eyebox size of CRIS is independent of the pixel pitch of the SLM. Consequently, the eyebox expansion ratio decreases linearly as the pixel pitch decreases. Based on this relationship, it might be anticipated that CRIS would lose its practicality if an SLM with a pixel pitch approaching the wavelength were developed. Nevertheless, CRIS remains advantageous in terms of accessibility and cost-effectiveness, even under such circumstances. This is because a reduction in pixel pitch, assuming a fixed display resolution, leads to a decrease in the physical size of the SLM and its field of view. To preserve the field of view and enable eyebox expansion, the resolution must increase correspondingly, significantly raising SLM costs and computational costs.

### Experimental setup

The lasers with wavelengths of 638 nm, 515 nm, and 450 nm are coupled by the single mode fiber and the lasers become spatially incoherent due to the rotating diffuser. The holographic diffuser with a divergence angle of ±10^∘^ and transmission efficiency of 85% is adopted. The spatially incoherent light collimated by the lens is illuminated through the PBS. Since an amplitude-only SLM rotates the polarization of light by an amount proportional to the modulation, only the modulated pattern of light passes through the PBS. The amplitude-only modulator, IRIS-F55 (MAY Inc.) with 1920 × 1080 resolution and 6.3 μm pixel pitch, is used to modulate the light, but only 1080 × 1080 area is used in the optical reconstruction to minimize the noise from the limited numerical aperture. It operates in grayscale at 180 Hz, which corresponds to 60 Hz for color display as it processes RGB sequentially. The RGB lasers are synchronized with the SLM, with each color illuminating during its corresponding frame.

We implemented CRIS using an amplitude-only SLM because phase-only SLMs require 4f-filtering to achieve high image quality. While holographic displays using phase-only SLMs can achieve high-quality images, their image quality becomes lower than that of amplitude-only SLMs due to the pixelated structures when 4f-filtering is not applied^35^. Since CRIS uses numerous angles of incident light to expand the eyebox, blocking high-frequency components during 4f-filtering would also block most incident light and prohibit eyebox expansion.

Due to the lens after the SLM, the virtual image of the SLM is located at infinity, corresponding to 0 diopters. In contrast, the virtual image of the front plane is located 67 cm away from the pupil, corresponding to 1.5 diopters. The lens after the SLM forms the Fourier plane of the hologram and the aperture mounted on the motorized stage moves near the Fourier plane. As a result, the aperture emulates a moving iris of a human.

The calibration of the SLM is performed by assigning one value to the entire SLM and measuring the modulated intensity. After measuring the intensity, a fitted function of output-input is applied to the input value to correct the modulation. Although the LCoS modulation of the wavefield depends on the incident angle, the difference is negligible if the angle difference is not much larger than 10^∘^^36^. To expand the viewing angle to larger than 10^∘^, digital micromirror devices can be adopted to minimize the dependency of the modulation on the incident angle. The overall optical efficiency of the experimental setup is ~4%, limited by the oversized illumination area, scattering from the diffuser and other optical components, and the unused light at zero-value pixels on the amplitude-only SLM.

The main reason for observing only a 32-fold expansion experimentally, instead of the theoretical 1000-fold, is the limited numerical aperture of the optical system. To observe the 1000-fold eyebox expansion, the lens after the SLM must accommodate all the incident angles reflected by the SLM. In the experimental setup, we used an optical system with a numerical aperture of 0.3, which allowed only a small portion of the light to pass through the lens. By using an optical system with a numerical aperture of larger than 0.8, it would be possible to achieve the theoretical maximum.

### Pupil area dependency

**Fig. 6:**
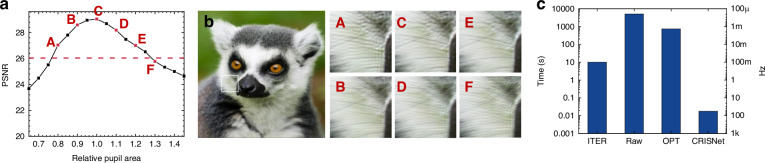
Pupil area dependency and computation time. **a** Average PSNR as a function of the relative pupil area. The PSNRs are evaluated on the DIV2K validation dataset. The red dashed line represents a—3dB line from the maximum PSNR. **b** Numerically reconstructed CRIS as a function of the relative pupil area. The large image represents the reconstructed CRIS at the relative pupil area of 1.3, corresponding to the point F in (**a**). The small images are enlarged versions, with the relative pupil areas of 0.8, 0.9, 1.0, 1.1, 1.2, and 1.3. Image quality degradation can be found in the enlarged images, while the difference is not noticeable in the overall images. **c** Computation time depending on the methods. “ITER” refers to the conventional iterative method with the gradient method^20,41^, “Raw” refers to the CRIS synthesis method without the optimized numerical reconstruction, and “OPT” refers to the CRIS synthesis method with the optimized numerical reconstruction

During the synthesis of the CRIS, only the propagated light passing through the pupil area ${\mathcal{A}}$ contributes to the reconstruction. Variations in the pupil area result in changes in the reconstructed intensity, leading to degradation of image quality (Fig. 6a). Considering that the pupil area depends on the brightness, the pupil area used in the synthesis can be calibrated by detecting ambient brightness. However, even under fixed ambient brightness, emotional arousal can cause variations in pupil area of up to ± 20% for a single individual^37,38^. According to the results of the numerical simulation, the variation in pupil area of ±20% can reduce the PSNR by 2 dB, which is not significant in most cases, given the transient nature of the variation. By estimating a 3 dB degradation in the PSNR, the FWHM of the relative pupil area is ~51%. In addition, interference and speckle-like patterns are not observed in the reconstructions with mismatched pupil areas because additional and subtractive light components are incoherent (Fig. 6b).

### Details of CRISNet

Real-time synthesis of the CRIS was achieved using the CRISNet and unsupervised training was adopted for the optimal image quality^39^. However, the numerical reconstruction of the CRIS requires 500 times more computational time than coherent propagation, making it an obstacle during training (Fig. 6c). The major computational challenge arises from the requirement to compute coherent propagations for a large number of different incident angles, with an expanded Fourier domain to accommodate off-axis propagations. Through optimization of the numerical reconstruction, such as employing a random selection of 100-300 incident angles instead of 400, along with cached tensors, the computation time can be reduced to 14.7%. CRISNet was trained by adopting the optimized reconstruction method, and the computation time was further reduced to 1/40000, enabling operation at 57 Hz on an NVIDIA RTX4090 without image quality degradation. The network was trained with a learning rate of 0.0001 for 100 epochs using the ADAM optimizer. The DIV2K training dataset^22^ was used to train the CRISNet, and each image was randomly cropped to a size of 1024 × 1024 to reduce the risk of overfitting. Moreover, during training, we observed a continuous increase in numerically reconstructed PSNRs synthesized from the DIV2K validation dataset, distinct from the DIV2K training dataset, indicating effective generalization and avoidance of overfitting. Since the DIV2K training dataset contains only 2D images, we generated random depth maps to synthesize multi-depth scenes. Each depth map is divided into an 11 × 11 grid, with each sub-patch in the grid assigned a uniform value randomly selected from a predefined range^40^.

**Fig. 7:**
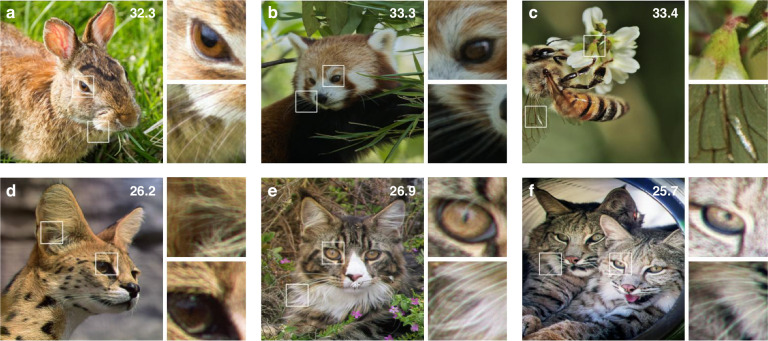
Reconstructed images of CRIS. Images in the Flickr2K dataset are numerically reconstructed (**a**–**c**) and optically reconstructed (**d**–**f**) when the location of the pupil is at the center of the eyebox. The PSNR in dB is marked in the top right corner, and the insets present enlarged images

**Fig. 8:**
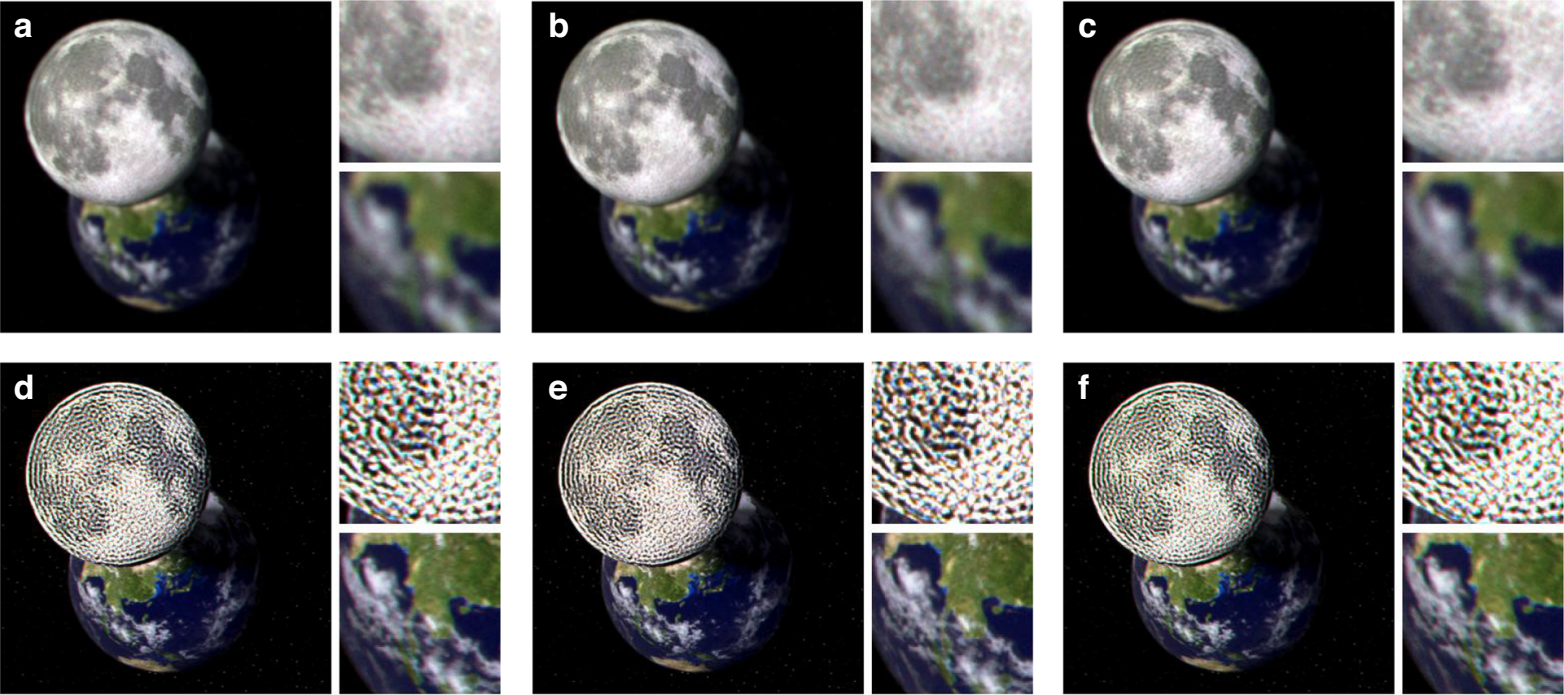
3D reconstruction at different pupil locations. The 3D scene is optically reconstructed for different pupil locations when the camera focus is on the front plane (**a**–**c**) and the SLM plane (**d**–**f**). Each pupil location of (**a**–**c**) corresponds to the points marked with the letters A, B, and C in Fig. 4e

CRISNet consists of 20 residual blocks, each containing two Conv2D layers, two leaky ReLU layers, and a shortcut connection. Each Conv2D layer has 32 input channels and 32 output channels, except for the first and last layers. The first layer has 4 input channels, and the last layer has 3 output channels, corresponding to the input and output channels of the network (Figs. 7 and 8).

## Supplementary information

Supplementary information

## Data Availability

All data needed to evaluate the conclusions in the paper are present in the paper and/or the Supplementary Materials.

## References

[1] Gabor, D. A new microscopic principle. *Nature* **161**, 777–778 (1948). 18860291 10.1038/161777a0

[2] Erf, R. *Holographic Nondestructive Testing* (Elsevier, 2012).

[3] Ashley, J. et al. Holographic data storage technology. *IBM J. Res. Dev.* **44**, 341–368 (2000).

[4] Park, Y., Depeursinge, C. & Popescu, G. Quantitative phase imaging in biomedicine. *Nat. Photonics* **12**, 578–589 (2018).

[5] Poon, T.-C. *Digital Holography and Three-Dimensional Display: Principles And Applications* (Springer Science & Business Media, 2006).

[6] Kim, M. K. & Kim, M. K. *Digital Holographic Microscopy* (Springer, 2011).

[7] Xu, W., Jericho, M., Meinertzhagen, I. & Kreuzer, H. Digital in-line holography for biological applications. *Proc. Natl Acad. Sci. USA* **98**, 11301–11305 (2001). 11572982 10.1073/pnas.191361398 PMC58724

[8] Javidi, B. & Tajahuerce, E. Three-dimensional object recognition by use of digital holography. *Opt. Lett.* **25**, 610–612 (2000). 18064126 10.1364/ol.25.000610

[9] Liu, J.-P., Tahara, T., Hayasaki, Y. & Poon, T. -C. Incoherent digital holography: a review. *Appl. Sci.* **8**, 143 (2018).

[10] Tahara, T. Review of incoherent digital holography: applications to multidimensional incoherent digital holographic microscopy and palm-sized digital holographic recorder-holosensor. *Front. Photonics* **2**, 829139 (2022).

[11] Rosen, J. & Brooker, G. Digital spatially incoherent fresnel holography. *Opt. Lett.* **32**, 912–914 (2007). 17375151 10.1364/ol.32.000912

[12] Poon, T.-C. Optical scanning holography-a review of recent progress. *J. Opt. Soc. Korea* **13**, 406–415 (2009).

[13] Vijayakumar, A. & Rosen, J. Interferenceless coded aperture correlation holography–a new technique for recording incoherent digital holograms without two-wave interference. *Opt. Express* **25**, 13883–13896 (2017). 28788831 10.1364/OE.25.013883

[14] Peng, Y., Choi, S., Kim, J. & Wetzstein, G. Speckle-free holography with partially coherent light sources and camera-in-the-loop calibration. *Sci. Adv.* **7**, eabg5040 (2021). 34767449 10.1126/sciadv.abg5040 PMC8589315

[15] Lee, S. et al. Light source optimization for partially coherent holographic displays with consideration of speckle contrast, resolution, and depth of field. *Sci. Rep.* **10**, 18832 (2020). 33139826 10.1038/s41598-020-75947-0 PMC7606540

[16] Kuo, G., Schiffers, F., Lanman, D., Cossairt, O. & Matsuda, N. Multisource holography. *ACM Trans. Graph.* **42**, 1–14 (2023).

[17] Ghoushchi, V. P., Aas, M., Ulusoy, E. & Ürey, H. Effect of spatial coherence of led sources on image resolution in holographic displays. in *Advances in Display Technologies VII* Vol. 10126, 63–70 (SPIE, 2017).

[18] Blanche, P.-A. Holography, and the future of 3d display. *Light. Adv. Manuf.* **2**, 446–459 (2021).

[19] Chang, C., Bang, K., Wetzstein, G., Lee, B. & Gao, L. Toward the next-generation vr/ar optics: a review of holographic near-eye displays from a human-centric perspective. *Optica* **7**, 1563–1578 (2020). 34141829 10.1364/OPTICA.406004 PMC8208705

[20] Yang, D. et al. Diffraction-engineered holography: beyond the depth representation limit of holographic displays. *Nat. Commun.* **13**, 1–11 (2022). 36224198 10.1038/s41467-022-33728-5 PMC9556550

[21] Kavaklí, K., Itoh, Y., Urey, H. & Akşit, K. Realistic defocus blur for multiplane computer-generated holography. In *Proc. IEEE Conference Virtual Reality and 3D User Interfaces (VR)* 418–426 (IEEE, 2023).

[22] Agustsson, E. & Timofte, R. Ntire 2017 challenge on single image super-resolution: dataset and study. In *The IEEE Conference on Computer Vision and Pattern Recognition (CVPR) Workshops* (IEEE, 2017).

[23] Kuo, G., Waller, L., Ng, R. & Maimone, A. High resolution étendue expansion for holographic displays. *ACM Trans. Graph.* **39**, 66–1 (2020).

[24] Tseng, E. et al. Neural étendue expander for ultra-wide-angle high-fidelity holographic display. *Nat. Commun.* **15**, 2907 (2024). 38649369 10.1038/s41467-024-46915-3 PMC11035703

[25] Park, J., Lee, K. & Park, Y. Ultrathin wide-angle large-area digital 3d holographic display using a non-periodic photon sieve. *Nat. Commun.* **10**, 1–8 (2019). 30898998 10.1038/s41467-019-09126-9 PMC6428928

[26] Gopakumar, M. et al. Full-colour 3d holographic augmented-reality displays with metasurface waveguides. *Nature* **629**, 791–797 (2024). 10.1038/s41586-024-07386-0 PMC11111399 38720077

[27] Kim, J. et al. Foveated ar: dynamically-foveated augmented reality display. *ACM Trans. Graph.* **38**, 99–1 (2019).

[28] Yaraş, F., Kang, H. & Onural, L. State of the art in holographic displays: a survey. *J. Disp. Technol.* **6**, 443–454 (2010).

[29] Liu, T. et al. Research progress of large viewing-area 3d holographic near-eye display. *Laser Photonics Rev.* **18**, 2300641 (2023).

[30] Stein, N. et al. A comparison of eye tracking latencies among several commercial head-mounted displays. *i-Percept.* **12**, 2041669520983338 (2021). 10.1177/2041669520983338 PMC7883159 33628410

[31] Shigematsu, O., Naruse, M. & Horisaki, R. Computer-generated holography with ordinary display. *Opt. Lett.* **49**, 1876–1879 (2024). 38621028 10.1364/OL.516005

[32] Maimone, A., Georgiou, A. & Kollin, J. S. Holographic near-eye displays for virtual and augmented reality. *ACM Trans. Graph.* **36**, 1–16 (2017).

[33] Zhao, Y., Cao, L., Zhang, H., Kong, D. & Jin, G. Accurate calculation of computer-generated holograms using angular-spectrum layer-oriented method. *Opt. Express* **23**, 25440–25449 (2015). 26480062 10.1364/OE.23.025440

[34] Lee, B., Kim, D., Lee, S., Chen, C. & Lee, B. High-contrast, speckle-free, true 3d holography via binary CGH optimization. *Sci. Rep.* **12**, 1–12 (2022). 35181695 10.1038/s41598-022-06405-2 PMC8857227

[35] Yang, D. & Lee, H. -S. Direct amplitude-only hologram realized by broken symmetry. *Sci. Adv.* **10**, eadp1205 (2024). 39213363 10.1126/sciadv.adp1205 PMC11364108

[36] Lizana, A. et al. Influence of the incident angle in the performance of liquid crystal on silicon displays. *Opt. Express* **17**, 8491–8505 (2009). 19434183 10.1364/oe.17.008491

[37] Partala, T. & Surakka, V. Pupil size variation as an indication of affective processing. *Int. J. Hum. Comput. Stud.* **59**, 185–198 (2003).

[38] Oliva, M. & Anikin, A. Pupil dilation reflects the time course of emotion recognition in human vocalizations. *Sci. Rep.* **8**, 4871 (2018). 29559673 10.1038/s41598-018-23265-x PMC5861097

[39] Shimobaba, T. et al. Deep-learning computational holography: a review. *Front. Photonics* **3**, 8 (2022).

[40] Yu, H. et al. Deep learning-based incoherent holographic camera enabling acquisition of real-world holograms for holographic streaming system. *Nat. Commun.* **14**, 3534 (2023). 37316495 10.1038/s41467-023-39329-0 PMC10267150

[41] Chakravarthula, P., Peng, Y., Kollin, J., Fuchs, H. & Heide, F. Wirtinger holography for near-eye displays. *ACM Trans. Graph.* **38**, 1–13 (2019).

