# Supplementary material for: Spatial incoherence-driven optical reconstruction of holograms with observer shift-invariance
Source: Light Sci Appl. 2025 May 12;14:191. doi: 10.1038/s41377-025-01823-z (PMC12069550; doi:10.1038/s41377-025-01823-z)
Supplement: Supplementary file 1 — Supplementary information [file 41377_2025_1823_MOESM1_ESM.pdf]

Supplementary material

Eyebox size of conventional holograms

Comparing the field of view (FoV), eyebox, and etendue between systems is a complex task, especially when considering the different light sources: coherent and incoherent. For simplicity, we compared a coherent holographic display system to the CRIS system, assuming that both systems have identical lenses and use the same SLM. In a coherent holographic display system, the relationship between the FoV and the eyebox limited by diffraction angle of the SLM is determined by the following equation,

$$\text{eyebox} = \frac{\lambda N}{2\sin(\text{FoV}/2)} = \frac{f\lambda}{\Delta x} \tag{S1}$$

where, f is the focal length of the lens after the SLM, Δx is the pixel pitch of the SLM, λ is the wavelength of the light and N is the number of pixels of the SLM. Since the eyebox of the coherent holographic display system is determined under fixed focal length, wavelength, and pixel pitch, it is possible to compare the eyebox values of the coherent holographic display system and the CRIS system using the parameters of the CRIS system. For each wavelength used in the experiment, the lengths of eyebox along radial direction are 1.5, 1.7, and 2.1 mm. Since the overall eyebox would be limited by the smallest size, the radial length of the eyebox would be 1.5 mm.

However, the eyebox size determined by the diffraction limit corresponds to the point where the reconstructed image completely disappears. Since the eyebox size of CRIS is determined by the Full Width at Half Maximum(FWHM) of the signal to noise ratio, corresponding to 3 dB degradation in the PSNR, the eyebox size of the conventional holograms should be calculated by using image quality for the fair comparison. The eyebox estimated by image quality can be calculated by assuming that the pupil is located on the Fourier plane (Fig.S1**a**). Under a spatially coherent light source, high-frequency components will be partially blocked by the pupil when the pupil shifts from the center. The blocked components result in a decrease in image quality, and the FWHM of image quality can be determined at the point where the average PSNR decreases by 3 dB (Fig.S1**b**). According to numerical calculation, the radial eyebox length is approximately 1.2 mm.

Similarly as in the manuscript, the length of the eyebox along the z-axis can be calculated by considering the blocked area when the pupil shifts along the z-axis (Fig.S1**c**). The half-length of the eyebox along the z-axis is given by $\frac{2}{\text{FoV}}p_r$, where p_r is the radial eyebox length. In the manuscript, the eyebox size estimated by image quality is used in comparison with the CRIS.

Generalization of shift-invariance along z-axis

By assuming the Fourier transformed field on the SLM plane as $\bar{U}(k_x, k_y, 0)$, the field at retina can be expressed as, (Fig. S2)

$$\begin{aligned} U(x'', y'', f+f+p_z+f') = \frac{H_0(2f+p_z+f')}{(2\pi)^5} \int\int e^{-if'\frac{k_x''^2+k_y''^2}{2k}} \int\int e^{-ik\frac{x'^2+y'^2}{2f'}} \int\int e^{-i(f+p_z)\frac{k_x'^2+k_y'^2}{2k}} \\ \times \int\int e^{-ik\frac{x^2+y^2}{2f}} \int\int e^{-if\frac{k_x^2+k_y^2}{2k}} \bar{U}(k_x, k_y, 0)\, e^{ik_x x + ik_y y} dk_x dk_y e^{-ik_x' x - ik_y' y} dx dy \\ \times e^{ik_x' x' + ik_y' y'} dk_x' dk_y' e^{-ik_x'' x' - ik_y'' y'} dx' dy' e^{ik_x'' x'' + ik_y'' y''} dk_x'' dk_y'', \end{aligned} \tag{S2}$$

where $H_0(d) = e^{-ikd}$ and the coordinates follow the marks of Fig. 5. Equation S2 is derived under the assumption that the field undergoes the following steps.

1. Propagate for a distance f
2. Phase altered by a lens ($e^{-ik\frac{x^2+y^2}{2f}}$)
3. Propagate for a distance $f + p_z$

4. Phase altered by an eye lens ($e^{-ik\frac{x'^2+y'^2}{2f'}}$)

5. Propagate for a distance f'

During the steps, Fourier transforms and inverse Fourier transforms are applied to the field.

In order to simplify the equation, the following formula would be used.

$$\begin{aligned}\iint e^{ia\left(x^2+y^2\right)}e^{ibx+icy}dxdy &= \iint e^{ia\left(x^2+\frac{b}{a}x+\left(\frac{b}{2a}\right)^2+y^2+\frac{c}{a}y+\left(\frac{c}{2a}\right)^2\right)}e^{-ia\left(\left(\frac{b}{2a}\right)^2+\left(\frac{c}{2a}\right)^2\right)}dxdy \\ &= e^{-i\frac{b^2+c^2}{4a}}\iint e^{ia\left(x^2+y^2\right)}dxdy \equiv C\left(a\right)e^{-i\frac{b^2+c^2}{4a}}\end{aligned} \tag{S3}$$

By applying Eq. S3 twice, Eq. S2 can be simplified as,

$$\begin{aligned}U\left(x'',y'',f+f+p_z+f'\right) &= \frac{H_0\left(2f+p_z+f'\right)}{(2\pi)^5}\iiiint\iiiint\iint e^{-if'\frac{k_x''^2+k_y''^2}{2k}} \\ &\times e^{-ik\frac{x'^2+y'^2}{2f'}}e^{-i(f+p_z)\frac{k_x'^2+k_y'^2}{2k}}e^{-ik\frac{x^2+y^2}{2f}}e^{-if\frac{k_x^2+k_y^2}{2k}}e^{ik_xx+ik_yy}e^{-ik_x'x-ik_y'y}e^{ik_x'x'+ik_y'y'}e^{-ik_x''x'-ik_y''y'} \\ &\times e^{ik_x''x''+ik_y''y''}\bar{U}\left(k_x,k_y,0\right)dk_xdk_ydk_x'dk_y'dk_x''dk_y''dxdydx'dy' \\ &= \frac{H_0\left(2f+p_z+f'\right)}{(2\pi)^5}\iiiint\iiiint e^{-if'\frac{k_x''^2+k_y''^2}{2k}}e^{-ik\frac{x'^2+y'^2}{2f'}}e^{-i(f+p_z)\frac{k_x'^2+k_y'^2}{2k}}e^{-if\frac{k_x^2+k_y^2}{2k}}C\left(-\frac{k}{2f}\right) \\ &\times e^{i\frac{\left(k_x-k_x'\right)^2+\left(k_y-k_y'\right)^2}{2k/f}}e^{ik_x'x'+ik_y'y'}e^{-ik_x''x'-ik_y''y'}e^{ik_x''x''+ik_y''y''}\bar{U}\left(k_x,k_y,0\right)dk_xdk_ydk_x'dk_y'dk_x''dk_y''dx'dy' \\ &= \frac{H_0\left(2f+p_z+f'\right)}{(2\pi)^5}\iiiint\iint e^{-if'\frac{k_x''^2+k_y''^2}{2k}}e^{-i(f+p_z)\frac{k_x'^2+k_y'^2}{2k}}e^{-if\frac{k_x^2+k_y^2}{2k}}e^{ik_x''x''+ik_y''y''}C\left(-\frac{k}{2f}\right) \\ &\times e^{i\frac{\left(k_x-k_x'\right)^2+\left(k_y-k_y'\right)^2}{2k/f}}C\left(-\frac{k}{2f'}\right)e^{i\frac{\left(k_x'-k_x''\right)^2+\left(k_y'-k_y''\right)^2}{2k/f'}}\bar{U}\left(k_x,k_y,0\right)dk_xdk_ydk_x'dk_y'dk_x''dk_y''.\end{aligned} \tag{S4}$$

Since Eq. S4 has only first order in terms of k_x'' and k_y'', we can replace the integral with δ function, *i.e.* $\iint e^{ik_x''x''+ik_y''y''}e^{i\frac{-2k_x'k_x''-2k_y'k_y''}{2k/f'}}dk_x''dk_y'' = (2\pi)^2\delta(x''-k_x'f'/k)\delta(y''-k_y'f'/k)$. Equation S4 would be simplified as,

$$\begin{aligned}&U\left(x'',y'',f+f+p_z+f'\right) \\ &= \frac{H_0\left(2f+p_z+f'\right)}{(2\pi)^3}C\left(-\frac{k}{2f}\right)C\left(-\frac{k}{2f'}\right)\iint e^{-i(f+p_z)\frac{\left(\frac{x''k}{f'}\right)^2+\left(\frac{y''k}{f'}\right)^2}{2k}}e^{-if\frac{k_x^2+k_y^2}{2k}} \\ &\times e^{i\frac{\left(k_x-\left(\frac{x''k}{f'}\right)\right)^2+\left(k_y-\left(\frac{y''k}{f'}\right)\right)^2}{2k/f}}e^{i\frac{\left(\frac{x''k}{f'}\right)^2+\left(\frac{y''k}{f'}\right)^2}{2k/f'}}\bar{U}\left(k_x,k_y,0\right)dk_xdk_y \\ &= \frac{H_0\left(2f+p_z+f'\right)}{(2\pi)^3}C\left(-\frac{k}{2f}\right)C\left(-\frac{k}{2f'}\right)e^{-i(f+p_z)\frac{\left(\frac{x''k}{f'}\right)^2+\left(\frac{y''k}{f'}\right)^2}{2k}}e^{i\frac{\left(\frac{x''k}{f'}\right)^2+\left(\frac{y''k}{f'}\right)^2}{2k/f'}}e^{i\frac{\left(\frac{x''k}{f'}\right)^2+\left(\frac{y''k}{f'}\right)^2}{2k/f}} \\ &\times \iint e^{-ik_xx''\left(\frac{f}{f'}\right)-ik_yy''\left(\frac{f}{f'}\right)}\bar{U}\left(k_x,k_y,0\right)dk_xdk_y \\ &= \frac{1}{\mathcal{N}_0}\iint e^{-ik_xx''\left(\frac{f}{f'}\right)-ik_yy''\left(\frac{f}{f'}\right)}\bar{U}\left(k_x,k_y,0\right)dk_xdk_y,\end{aligned} \tag{S5}$$

where, $\mathcal{N}_0$ includes all phase factors which would be ignored during the intensity calculation. The last line of Eq. S5 is the same as Eq. M8.

Number of incident angles

To numerically simulate incoherent light propagation, coherent propagation is calculated for all incident angles and all the coherent propagation results are incoherently added. Ideally, an infinite Number of Incident Angles (NoIA) would be required to numerically simulate incoherent light. However, 400 NoIA offer similar results to those of the

much larger numbers, and thus we selected 400 as the standard NoIA (Fig. S3**a**). More specifically, we divided the 2-dimensional area of NoIA into a regular grid, ensuring that each axis has an equal number of divisions.

To reduce the optimization time of the CRIS, we randomly selected the NoIA. First, we selected a prime number as a difference between the minimum and the maximum of the range to maximize the effect of selecting different numbers. Among them, 2 and 3 seemed insufficient, and 5 was avoided since it is a multiple of 10. Thus, we set 7 as the difference between the minimum and maximum. Subsequently, to find the optimal value of the minimum, we synthesized a CRIS using the NoIA range from x to $x+7$, where x can be from 5 to 14 (Fig. S3**b**). To analyze the synthesized CRIS depending on the NoIA range, we evaluated the PSNR of the synthesized CRIS by using 400 NoIA. We selected $x = 10$ among them, because it seems sufficient to provide a reasonable PSNR while providing sufficient speed.

Wavelength dependency of CRIS

While the manuscript assumes temporal coherence in CRIS reconstruction, it is noteworthy that the CRIS can be successfully reconstructed even in the presence of temporal incoherence, provided that the spectral bandwidth is not excessively broad. Figure S4 depicts the average PSNR as a function of the wavelength difference between the wavelength used in synthesis and the wavelength used in reconstruction. With a wavelength difference as large as 100 nm, the degradation in the PSNR is only approximately 0.5 dB. Therefore, it is anticipated that the reconstructed image quality with LED light sources would experience minimal degradation.

Additional loss to enhance defocus blur

To demonstrate the enhancement of defocus blur, we adopt the following defocus blur loss function,

$$\mathcal{L}_{\mathrm{DB}} = -\alpha\left|\frac{\nabla I(x,y)}{I(x,y)+1}\right| + \beta|\nabla I_G(x,y;\sigma)|, \tag{S6}$$

where, $I(x,y)$ is the numerically reconstructed intensity of the CRIS at the SLM plane, $I_G(x,y;\sigma)$ is the Gaussian blur intensity with the sigma value σ, and $\alpha(\beta)$ is a user-selected constant. The first term of Eq. S6 enhances speckles similar to phase-only holograms. At the same time, the second term improves defocus blur by reducing the overall gradient while excluding speckle patterns through Gaussian blur. Figure S5 presents reconstructed images with adopting the defocus blur loss, where $\sigma = 1$, $\alpha = 1$, $\beta = 0.005$. The demonstrated loss function is one example of additional loss functions to enhance defocus blur, and it is anticipated that natural defocus blur can be achieved through further research.

Comparison with incoherent digital holography

CRIS is similar to incoherent digital holography methods including FINCH and I-COACH in that it uses an incoherent light source. However, the key difference lies in their purposes: incoherent digital holography methods are designed for holographic data acquisition, whereas CRIS is an optical reconstruction method for displays.

Specifically, we can compare CRIS and I-COACH in detail, as CRIS resembles I-COACH in its lack of two-wave interference. In I-COACH, light distributions are captured at the camera sensor by displaying different patterns on the SLM. The captured data is then converted into a hologram by calculating the cross-correlation between object holograms and point spread holograms. This process requires more than two patterns and involves post-processing of the captured data to compute the cross-correlation.

In contrast, using multiple patterns and relying on post-processing are not feasible in holographic displays because the incoherent light passing through the SLM directly reaches the eye of an observer. To optically reconstruct CRIS without multiple patterns and post-processing, we utilize the pupil aperture of the eye. After decomposing the incoherent light into coherent light, we accounted for the masking effect of the pupil aperture for each coherent light. Due to the pupil, each modulated coherent light becomes different, and the incoherent sum of the modulated light can be optimized to reconstruct the desired 3D scene. In summary, CRIS differs from I-COACH by enabling single-shot reconstruction and eliminating the need for post-processing, thanks to its use of the pupil aperture.

Incoherent light and image quality

Theoretically, holograms reconstructed using incoherent light sources exhibit lower image quality compared to those with coherent light sources. However, under non-ideal experimental conditions, the difference in image quality is negligible, and in some cases, coherent light holograms may even have lower image quality. The reduction is primarily due to speckle noise and diffraction patterns caused by dust. In contrast, such noise is significantly suppressed in holograms with incoherent light sources. The suppression occurs because incoherent light is a sum of light from various angles, and the apparent position of dust is different from angle to angle on the object plane.

Figure S7 illustrates numerical simulations of reconstructed holograms with incoherent and coherent light sources. As the number of dust particles increases, diffraction patterns from the dust become more noticeable under coherent light sources (Fig. S7**a-c**). In contrast, negligible changes are observed with incoherent light sources (Fig. S7**d**). To assess the effects of dust, we synthesized and reconstructed a coherent hologram with high image quality[1]. Under the ideal condition, the conventional hologram presents high image quality with a PSNR of 47 dB (Fig. S7**e**). However, the presence of 40 dust particles in the optical system reduces the reconstructed image quality, decreasing the PSNR by 15 dB (Fig. S7**f**). In contrast, in CRIS, image quality degradation is negligible due to the incoherent light, resulting in higher image quality compared to the coherent hologram (Fig. S7**h**).

Image quality of coherent holograms can be improved through camera-in-the-loop (CITL) techniques, which optimize the hologram by capturing optically reconstructed intensities. However, CITL requires calibration for each device, and recalibration is necessary whenever dust positions change. Therefore, the inherent noise suppression of incoherent light provides an advantage in maintaining image quality in practical environments.

Performance evaluation of lightweight networks

Most neural networks for holographic displays, including CRISNet, operate in real time on high-performance hardware but slow down significantly when implemented on portable devices. From the perspective of accessibility and cost-effectiveness, it could be challenging to use such hardware for every holographic display. To address the issue, there could be two possible solutions.

1. Perform computations externally using cloud servers or local servers.
2. Develop a highly lightweight deep learning model for on-device computation.

The rapid improvement in on-device AI performance suggests that the second option could also be viable. However, lightweight networks may suffer from reduced performance. To assess the performance, we trained models with a reduced number of residual blocks.

Figure S9 presents the PSNR of the reconstructed intensities of each model, while the number of residual blocks of each model is 20, 10, and 5. The model we used in the manuscript has 20 residual blocks. The PSNR difference between the 20-residual-block model and the 5-residual-block model is 0.8 dB, while the latter model only requires one-quarter of the computation time. Since the 5-residual-block model requires 1.06×10^{12} operations for each 1920×1080 resolution image, it is theoretically possible to realize real-time processing with a 30 TOPS (tera operations per second) neural processing unit (NPU), which can be achieved with recently developed NPUs. While achieving the maximum TOPS would require adjusting the model structure to better align with the hardware architecture, real-time processing is anticipated to become feasible in the near future.

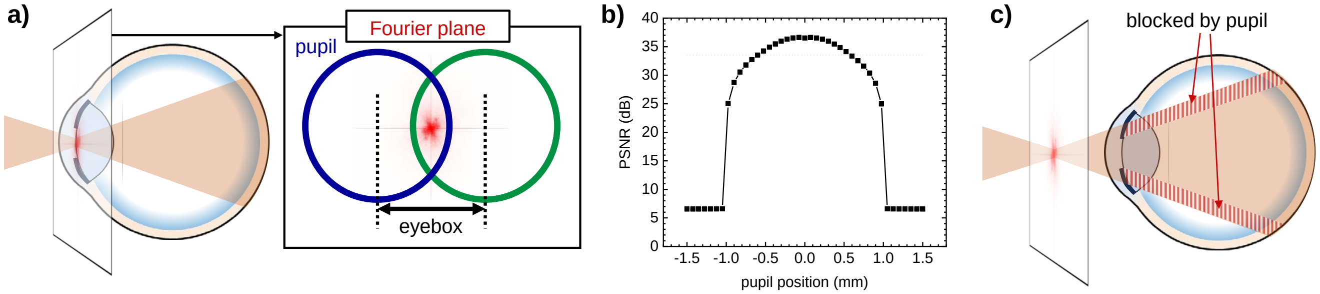

FIG. S1: **Eyebox calculation of conventional holograms. a,** Intensity of Fourier transformed hologram and pupil. If the shifted distance of the pupil is similar to the radius of the pupil, holograms are partially blocked. **b,** PSNR as a function of the pupil position. The diameter of the pupil is assumed to be 2 mm. The holograms are synthesized by the DIV2K validation dataset by ASM, and are assumed to be played with an ideal complex SLM. **c,** Blocked hologram due to the shift along the z-axis.

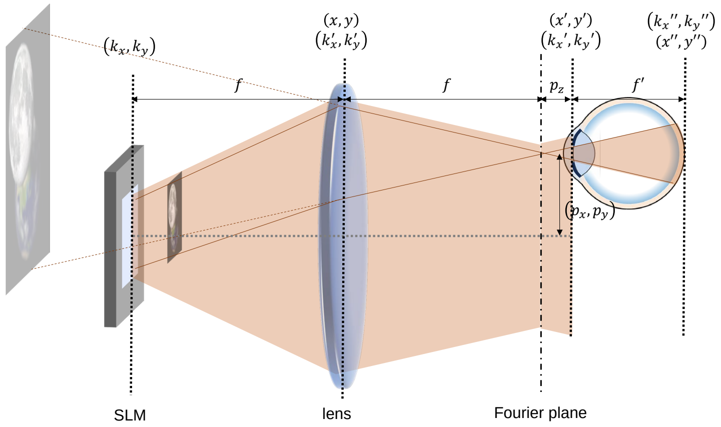

FIG. S2: **Schematics for calculating the generalization of shift-invariance along the z-axis.**

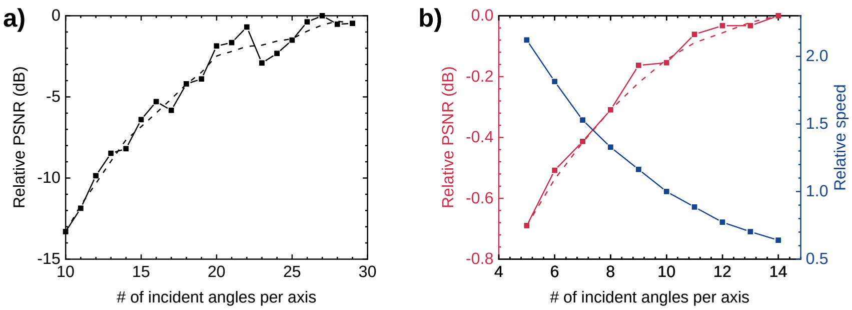

FIG. S3: **Relative PSNR as a function of NoIA. a,** Relative PSNR of the numerical reconstruction as a function of NoIA per axis. The dashed line represents the values smoothed by the adjacent-averaging method. **b,** Relative PSNR of the synthesized CRIS as a function of the minimum NoIA per axis.

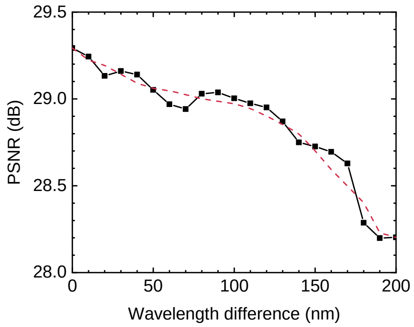

FIG. S4: **PSNR as a function of wavelength difference.** The wavelength difference refers to the variance between the wavelength utilized in synthesis and that utilized in reconstruction for all three colors. The red dashed line represents the values smoothed by the adjacent-averaging method.

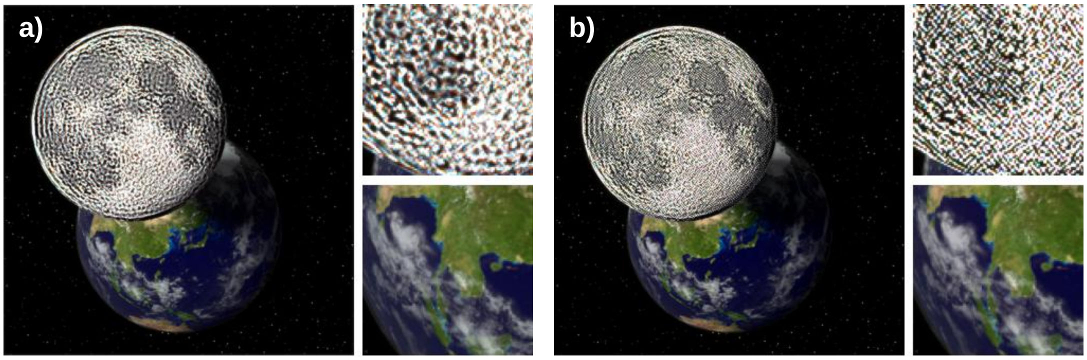

FIG. S5: **Numerically reconstructed intensities with the additional loss. a,** Numerically reconstructed intensity without $\mathcal{L}_{\mathrm{DB}}$, with the focus on the SLM plane. **b,** Numerically reconstructed intensity with $\mathcal{L}_{\mathrm{DB}}$, with the focus on the SLM plane.

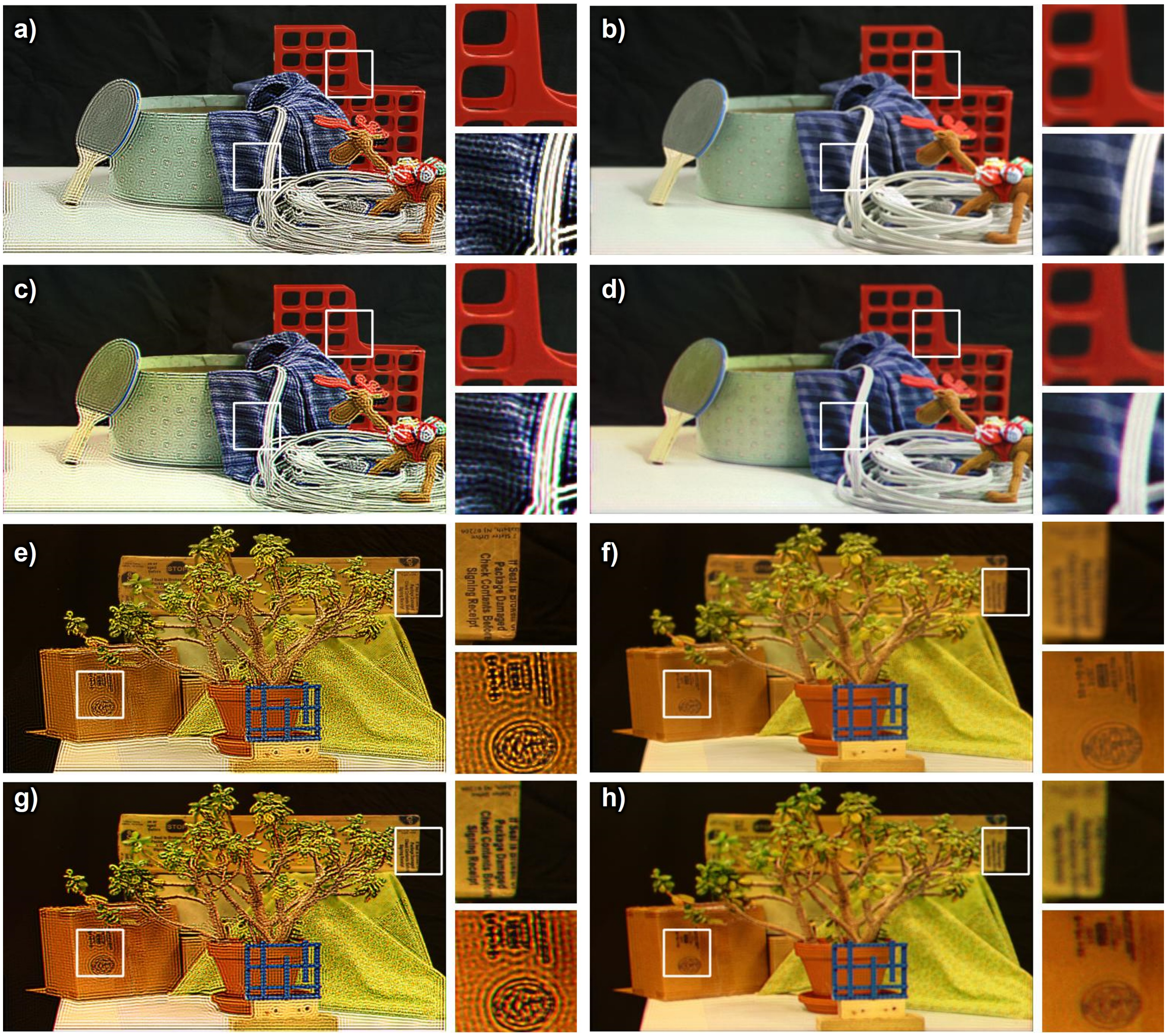

FIG. S6: **Numerically and optically reconstructed 3D scenes** Middleburry dataset[2] is used for reconstructing the 3D scenes. The camera focus is adjusted to the rear plane (**a**, **c**, **e**, **g**) and the front plane (**b**, **d**, **f**, **h**). Numerical reconstruction results (**a**, **b**, **e**, **f**) and optical reconstruction results (**c**, **d**, **g**, **h**) are nearly identical.

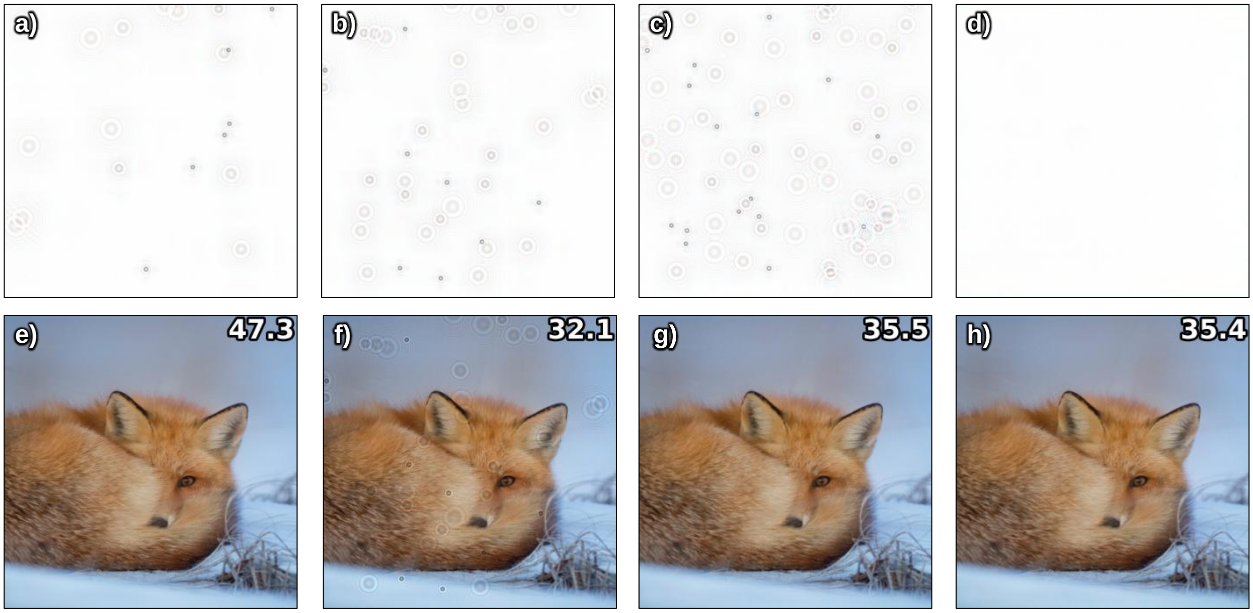

FIG. S7: **Numerically reconstructed holograms using coherent and incoherent light sources.** Diffraction patterns of dust under coherent light sources with varying numbers of dust particles are shown. The numbers of dust particles are 20, 40, and 80, corresponding to panels (**a**)-(**c**). Light distribution of an incoherent light source shows uniform white intensity under the condition of 80 dust particles (**d**). Numerically reconstructed coherent holograms with an ideal optical system (**e**) and a dusty system (**f**). Numerically reconstructed CRIS with the ideal optical system (**g**) and the dusty system (**h**). For the numerical simulation of the dusty system, the number of dust particles was assumed to be 40.

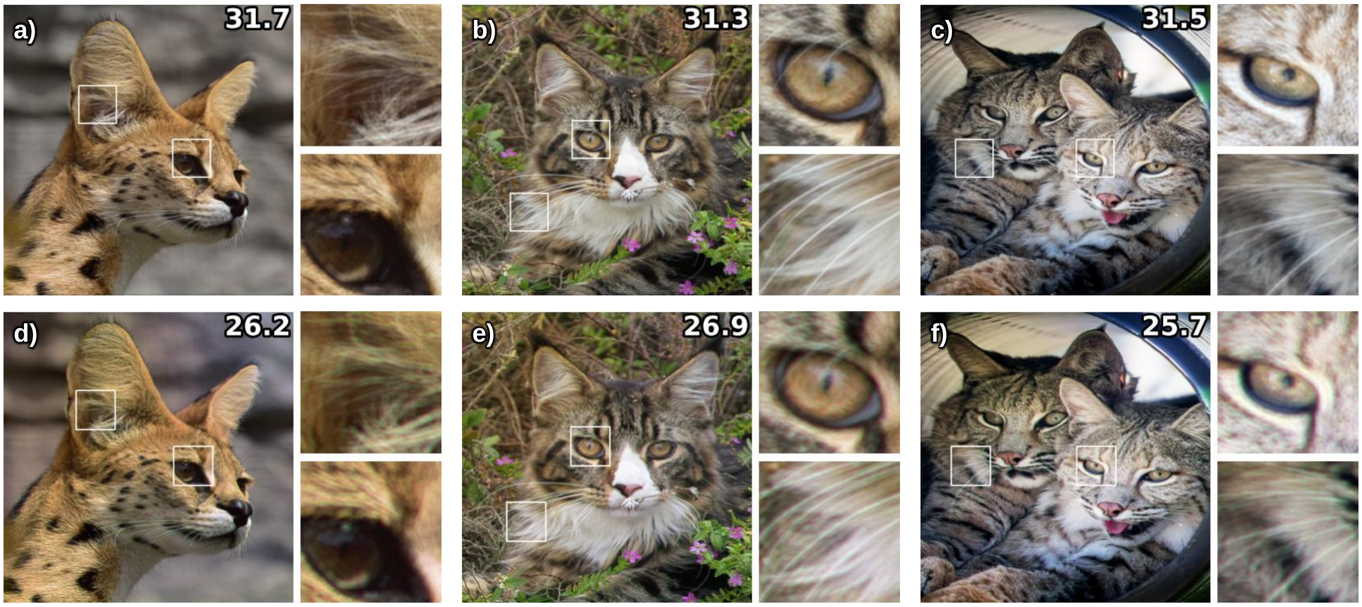

FIG. S8: **Comparison of numerically and optically reconstructed floating 2D scenes.** The numerically reconstructed intensities (**a**, **b**, **c**) present higher PSNRs than the optically reconstructed intensities (**d**, **e**, **f**), likely due to experimental imperfections such as optical aberrations and noise.

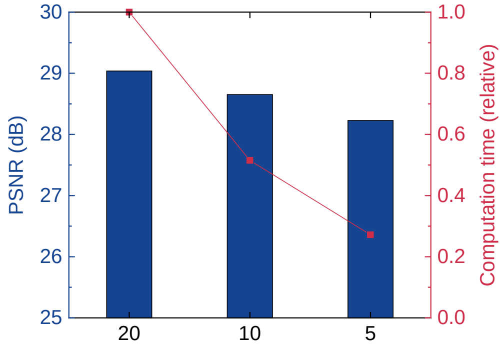

FIG. S9: **Image quality and computation time depending on the number of residual blocks.** The x-axis represents the number of residual blocks in the model. Bars indicate the PSNR of the models, while points represent the relative computation time.

REFERENCES

[1] A. Maimone, A. Georgiou, and J. S. Kollin, Holographic near-eye displays for virtual and augmented reality, ACM Transactions on Graphics (Tog) **36**, 1 (2017).

[2] D. Scharstein, H. Hirschmüller, Y. Kitajima, G. Krathwohl, N. Nešić, X. Wang, and P. Westling, High-resolution stereo datasets with subpixel-accurate ground truth, in *German Conference on Pattern Recognition* (Springer, 2014) pp. 31–42.
